# Impacts of Land‐Use Change on the Hydrology of Lake Tana Basin, Upper Blue Nile River Basin, Ethiopia

**DOI:** 10.1002/gch2.202200041

**Published:** 2022-06-11

**Authors:** Birhan Getachew, Busnur Rachotappa Manjunatha

**Affiliations:** ^1^ Geoinformatics Programme Department of Marine Geology Mangalore University Mangalagangothri Karnataka 574199 India; ^2^ Department of Geography and Environmental Studies Debre Tabor University P.O. Box 272 Ethiopia; ^3^ Department of Marine Geology Mangalore University Mangalagangothri Karnataka 574199 India

**Keywords:** hydrological response, lake tana basin, land use changes, supervised classification, SWAT

## Abstract

Human activities impact hydrology through changes in land use and land cover. This study examins the effects of changing land use on hydrological processes using the soil and water assessment tool (SWAT) model. The data is acquired from Landsat 4‐5 Thematic Mapper (TM) in 1989, Landsat 7 Enhanced Thematic Mapper Plus (ETM+) in 2005, and Landsat 8 Operational Land Inventory (OLI) in 2019. Image preprocessing, which includes georeferencing, radiometric and atmospheric correction, image enhancement, band composite, mosaicking, and sub‐setting, are performed. After that, supervised classification, accuracy assessment, and change detection are carried out. The hydrological changes in 1989, 2005, and 2019 are analyzed using land‐use maps. The SWAT model's calibration, validation, and sensitivity analysis are performed using the Integrated Parameter Estimation and Uncertainty Analysis Tool in the four main rivers of the basin. Farmlands and built‐up lands are found to have steadily increased in the basin, while shrublands, grasslands, and bare lands declined. Due to an expansion of agricultural and built‐up lands and a decrease in shrublands and grasslands, the basin's mean annual water yield and surface runoff increased in 2019, while evapotranspiration and lateral flow decreased compared to 1989 and 2005. Therefore, future watershed and basin management shall consider changing land use.

## Graphical abstract

The soil and water assessment tool (SWAT) model is used to assess the effects of land‐use change on the hydrological processes of the Lake Tana basin. According to the findings, the SWAT model is essential for effective modeling of the impact of land‐use change on water quality and quantity.

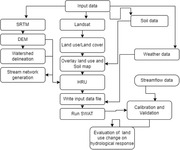

## 1 Introduction

Agriculture is the primary water user in Sub‐Saharan Africa, and food demand and water scarcity are increasing rapidly with the region's rapidly growing population.^[^ ^1^, ^2^ ^]^ Land‐use change is the primary cause of global societal challenges such as food security, climate change, and biodiversity loss.^[^ ^3^ ^]^ Climate change will significantly impact the natural water cycle and water supply. Changes in spatial‐temporal trends and precipitation fluctuations impact water supply replenishment.^[^ ^4^, ^5^ ^]^ Changes in land use and climate may have both immediate and long‐term effects on terrestrial hydrology affecting the balance of rainfall and evapotranspiration and the resulting runoff.^[^ ^6^, ^7^, ^8^, ^9^ ^]^

Changing land usage would necessitate a better understanding of the various processes that occur on the land.^[^ ^10^ ^]^ Changing land use, particularly in hydrological perspectives, has a considerable impact on surface runoff generation, infiltration, surface, and groundwater availability, and so on.^[^ ^11^, ^12^, ^13^, ^14^, ^15^, ^16^, ^17^ ^]^ Studies of rates, patterns, and implications of land use and land cover (LULC) dynamics at a watershed can pave the way to formulate and implement appropriate land management strategies and policies.^[^ ^3^, ^18^ ^]^

The degradation of natural resources that sustain human and ecosystem services on a global scale will intensify climate change and land use.^[^ ^19^ ^]^ Human activities alter hydrology, and the flow of water through ecosystems alteration over time.^[^ ^15^, ^20^, ^21^ ^]^ indicated that changes in land use eventually change the rainfall route to runoff by changing essential elements of hydrology, such as recharge of groundwater, surface runoff, interception, infiltration, evaporation, and percolation. There are several methods for determining the hydrological impacts of changing climate and land use. Approaches for estimating the hydrological effects of LULC alterations in river basins include paired catchments, multivariate statistics, and hydrological modeling. Different hydrological models have been developed to better understand the impact of soil properties and changing climate on hydrology and water resources.^[^ ^22^ ^]^ Therefore, hydrological models have become invaluable tools for dealing with changes in hydrological processes due to environmental factors.^[^ ^23^ ^]^ Various hydrological models are usable, depending on model inputs and parameters and the degree to which physical principles are applied.^[^ ^22^, ^24^ ^]^ For example, the variable infiltration capacity model (VIC) performs well in most stations, whereas the MIKE System Hydrologique Europeen model's applicability is limited because of its essential physical parameter and data requirements. On the other hand, TOPography‐based hydrological MODEL is appropriate for catchments with moderate topography and shallow soil.

Unlike the previously mentioned hydrological models, the soil and water assessment tool (SWAT) model requires only a tiny direct calibration to produce accurate hydrological predictions.^[^ ^22^, ^25^ ^]^ Furthermore,^[^ ^26^ ^]^ indicated that the SWAT model is a good estimator of hydro‐sedimentological processes that decision‐makers can use to manage water and environmental resources. To this effect, the SWAT model was applied in this investigation. A popular way to evaluate the influence of LULC alterations on hydrology is to utilize a physically dependent distributed hydrological model.^[^ ^15^ ^]^ This includes analyzing spatial hydrological responses to various LULC changes, evaluating temporal river flow responses to LULC changes, comparing basin‐scale mean values of simulated hydrological components in response to basin‐scale LULC changes, and evaluating temporal river flow responses to LULC changes.^[^ ^7^, ^27^, ^28^ ^]^

The Lake Tana basin is impacted by severe environmental destruction (deforestation, changing land use, soil erosion, flooding, contamination caused by misuse, and mismanagement of available natural resources.^[^ ^29^, ^30^ ^]^ The impact of changing land use on hydrology at various temporal and spatial scales has been studied in numerous studies.^[^ ^2^, ^20^, ^23^, ^31^, ^32^ ^]^ For instance,^[^ ^33^ ^]^ indicated that an increase in farmland and deforestation had caused a decrease in evapotranspiration and an increase in surface runoff in the Lake Tana basin. Another study by^[^ ^34^ ^]^ indicated that groundwater recharge reduced, while surface runoff increased in the Gilgel Abay watershed of the Lake Tana basin due to increased farmland. On the other extreme,^[^ ^35^ ^]^ stated that surface runoff decreased in 2001 compared to 1986 LULC but increased in 2015 compared to 2001 LULC in the Lake Tana basin. A similar study^[^ ^15^ ^]^ also described that the increase of cultivation land and decrease of woody shrub lands contribute to a decline in groundwater components and an increase in surface runoff in the Lake Tana basin.

On the other hand,^[^ ^36^ ^]^ indicated that an increase in the forest, plantation, and grassland area in Gibe catchment had increased the annual flow of the study area. Furthermore,^[^ ^37^ ^]^ found that land‐use change had a more significant impact on water quantity and quality than climate variability. Besides, in the future, land‐use change scenarios, yearly streamflow, and sediment load would increase by 0.19% to 0.45% and 0.22% to 0.68, respectively.^[^ ^38^ ^]^

Even though a substantial amount of research has been undertaken to analyze the impact of land‐use change on hydrological processes in the basin, many of the findings disagree on their results, signs, and extent of change. Thus, their results are not reproducible. As a result, more research is needed to develop acceptable water management strategies and optimize the utilization and management of available water resources. Furthermore, the effects of land‐use change on hydrological processes vary depending on the study area's geographic location. Thus, this study aimed to evaluate the hydrological response of the basin to the changing land use. The study's primary objective is to detect a land‐use change in the study area; to evaluate the effects of changing LULC on the hydrology of the Lake Tana basin.

## 2 Experimental Section

### 2.1 Description of the Study Area

**Figure 1:**
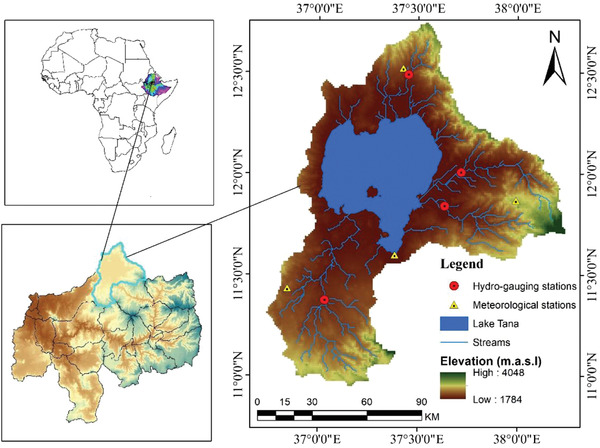
Map of the study area.

**Figure 2:**
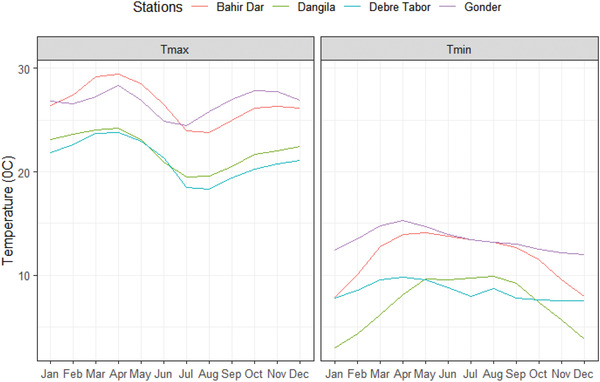
Mean monthly maximum and minimum temperature in four principal stations.

**Figure 3:**
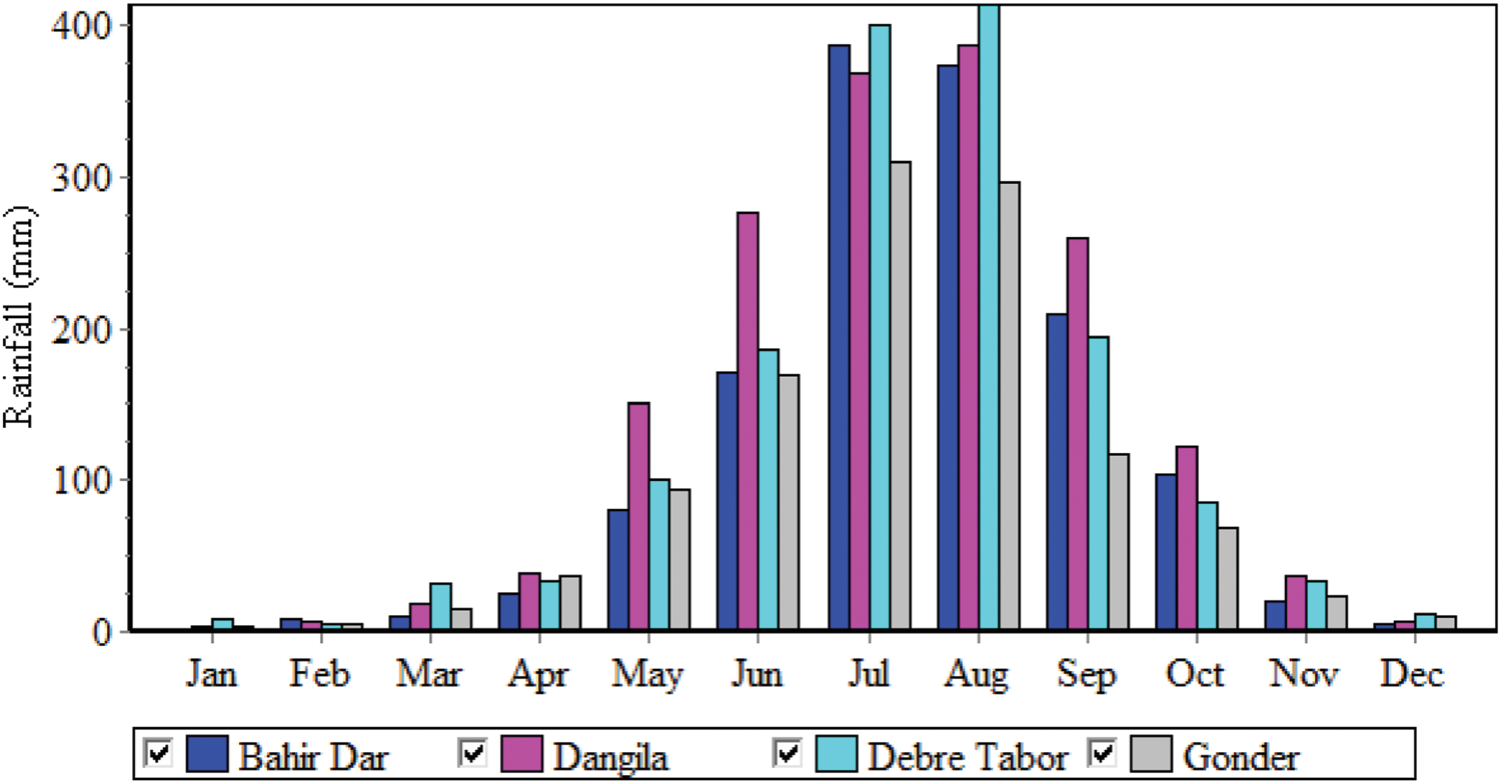
Total monthly rainfall in four principal stations.

The total area in the Lake Tana basin, including the Lake area, is around 15 096 km^2^ (**Figure** **1**). Geographically, the basin is located from 10°55 “to 12°45” N latitude and 36°40’ to 38°20’ E longitude.^[^ ^39^ ^]^ The estimated annual precipitation for the basin is ≈1280 mm. It is also estimated that the basin's mean yearly water yield and actual evapotranspiration are 392 and 773 mm, respectively. The basin's main rainy season is summer (Kiremet), which lasts from mid‐June to mid‐September, with the basin's climate being predominantly tropical highland monsoon. Similar to the region, with a mean annual temperature of about 20 °C, the air temperature shows high diurnal but slight yearly and seasonal temperature variation. Unimodal rainfall distribution occurred in the basin. **Figures** **2** and **3** show the mean monthly maximum and minimum temperature and rainfall in four principal stations. Lake Tana is the basin where the available water supplies utilize irrigation and hydroelectric power production. The Lake Tana basin has remarkable national significance. It encompasses high irrigation potential, such as Foggara and Debian plain, Tiss Abay, and Tana Beles hydroelectric power generation, high‐value crops, especially cash crops and livestock production, and ecotourism. Lake Tana, a natural reservoir located in this basin, is the main source of the Blue Nile River. It is ≈84 km long, and 66 km wide and is situated in the north‐western highlands of the country. The lake is a natural freshwater lake covering an area of 3000–3600 km^2^ at an altitude of 1800 m. It is a highland and shallow lake with a maximum depth of 15 m. The Megech, Ribb, Gumera, and Gilgel Abay rivers are Lake Tana's main tributaries, contributing higher than 93% of the annual water budget of the lake.

### 2.2 SWAT Model Inputs

#### 2.2.1 Digital Elevation Model (DEM)

The Shuttle Radar Topographic Mission (SRTM) 30 m data were accessed from the National Aeronautics and Space Administration (NASA; website https://dwtkns.com/srtm30m/). It has the highest resolution and lowest RMSE of 5.14 m in Addis Ababa compared to Advance Spaceborne Thermal Emission and Reflectance Radiometer (ASTER GDEM). DEM was used to classify the slopes and delineate the watersheds in the area.^[^ ^40^ ^]^ Therefore, SRTM DEM data were used to delineate the watershed boundary, SW (sub‐watershed), HRU (hydrological response units), and slope classification. The maximum number of slope classes in the SWAT models is up to five categories.^[^ ^41^ ^]^ Therefore, the slope was classified as (0–5%, 5–15%, 15–30%, and >30%) for the basin.

#### 2.2.2 Soil Data

**Figure 4:**
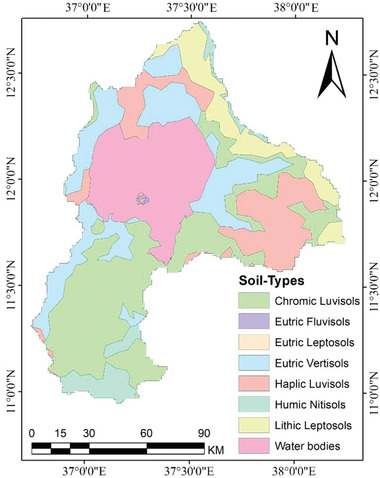
Major soil types in the Lake Tana basin.

The soil data used in this study came from the Ministry of Agriculture (MoA). Soil data were resampled to a spatial resolution of 30 m to have a similar resolution to elevation and land use data. The major soil types in the basin are Lithic Leptosols, Humic Nitisols, Chromic Luvisols, Eutric Fluvisols, Eutric Vertisols, Haplic Luvisols, and Waterbodies (**Figure** **4**).

#### 2.2.3 Land Use Land Cover

Landsat 4–5 Thematic Mapper (TM), Landsat 7 Enhanced Thematic Mapper (ETM), and Landsat 8 Operational Land Inventory (OLI) multitemporal satellite imagery were used in this work to map the changing pattern of land use and land cover in the study area. The basin was divided into seven land‐use classes. These classes of LULC were incorporated into the SWAT model.

#### 2.2.4 Hydro‐Meteorological Data

Maximum and minimum temperatures, rainfall, wind speed, relative humidity, and solar radiation are all required inputs for the SWAT model. The National Meteorological Agency (NMA) provided these data sets. Streamflow data for Ribb, Megech, Gumera, and Gilgel Abay were collected from Ethiopia's Ministry of Water, Irrigation, and Electricity (MOWIE). These data were used for model calibration and validation purposes. Hence, 70% and 30% of the data were used for calibration and validation. The SWAT model outputs were used to test whether the model reflects the essential observed water balance components (surface runoff) at gauging stations.

### 2.3 Soil and Water Assessment Tool Plus (SWAT+) Model Setup

The current streamflow, surface runoff, water yield, lateral flow, and evapotranspiration in the basin were assessed using the Soil and Water Assessment Tool (SWAT+) model. It allows for a more flexible spatial representation of watershed interactions and processes^[^ ^42^, ^43^ ^]^ Because the SWAT+ sub‐basins had two landscape units (i.e., floodplain and upslope). The total number of HRUs defined from SWAT+ was more extensive than those determined from SWAT.^[^ ^44^ ^]^ It operates on a daily time step. The model is used to predict the impact of land management strategies on water, sediment, and agricultural chemical yields in broad ungagged basins. The SWAT model can calculate total water yield, groundwater flow, surface runoff, percolation, and potential and actual evapotranspiration.^[^ ^45^ ^—^ ^47^ ^]^ The water balance equation of SWAT is

(1) $$\begin{array}{c} {\text{SW}}_{t}={\text{SW}}_{0}+\sum_{t=i}^{t}\left(R_{i}-Q_{i}-{\text{ET}}_{i}-P_{i}-{\text{QR}}_{I}\right) \end{array}$$

where (t) is the number of days, SW 0 (mm) is the beginning water content of the soil, SW t (mm) is the final water content of the soil, and P, QR, R, ET, and Q are the daily units of percolation, return flow, rainfall, evapotranspiration, and runoff, respectively; all amounts are in millimeters.

### 2.4 Model Calibration and Validation

SWAT models are increasingly utilized to support judgments on alternative management strategies in climate change, LULC change, pollution control, and water allocation.^[^ ^48^, ^49^, ^50^ ^]^ As a result, the model is subjected to a thorough uncertainty and calibration study. As a result, the integrated parameter estimation and uncertainty analysis tool (IPEAT) is used for model calibration and validation for the SWAT+ model. It is more flexible, easier to maintain, and more developed than the old SWAT model.^[^ ^51^, ^52^ ^]^ Streamflow data from the Gumera, Ribb, Gilgel Abay, and Megech river basins were used to calibrate and validate the model for the 1980–1999 and 2000–2007 time periods, respectively. The model performance assessment selected the parameters REVAL_MIN, REVAL_CO, FLOW_MIN, ALPHA, DELAY, ESCO, AWC, and CN2. Hence, sensitivity analysis of these parameters plays a crucial role in reducing parameter dimension and time required for model calibration.^[^ ^53^ ^]^ The hydrological parameter sensitive to model performance was calibrated using the sensitivity analysis method until a satisfactory agreement between the model‐simulated and observed values was obtained. Researchers working in un‐gauged basins with limited access to data on finer spatial scales can use the model because it only requires a few inputs. As a result, model outputs are only as accurate as the data and equations used to create them.^[^ ^54^ ^]^ Accord ing to (Arnold et al. 1998), the first stage in calibration is conducting a sensitivity analysis. After conducting sensitivity analysis, calibration was conducted using streamflow. The performance of the SWAT model is assessed using the percent of bias (PBIAS) correlation coefficient (*R* ^2^) and Nash Sutcliffe Efficiency (NSE). If the NSE value is around zero, the simulation cannot predict discharge, whereas an NSE of 1 shows that the model's performance is within an acceptable range of uncertainty. Any NSE value above 0.5 is good. The *R* ^2^ is the coefficient correlation between simulated and observed streamflow data (Equation (3), and values near one indicate the model's ability to predict observed values reliably. PBIAS quantifies the tendency of model values to be smaller (underestimation) or larger (overestimation) than actual values, with zero indicating the most accurate model simulation (Equation (4))

(2) $$\begin{array}{c} \text{NSE}=1-\frac{\sum_{t=1}^{n}\left(xi-yi\right)2}{\sum_{t=1}^{n}\left(xi-X_{\text{avg}}\right)2} \end{array}$$

(3) $$\begin{array}{c} R^{2}=\left[\frac{\sum_{i=1}^{n}\left(x_{i}-\bar{x}\right).\left(y_{i}-\bar{y}\right)}{\sqrt{\sum_{i=1}^{n}{\left(x_{i}-\bar{x}\right)}^{2}}.\sum_{i=1}^{n}{\left(y_{i}-\bar{y}\right)}^{2}}\right]2 \end{array}$$

(4) $$\begin{array}{c} PBIAS=\frac{\sum_{t=1}^{n}\left(xi-yi\right)}{\sum_{t=1}^{n}xi}\text{​} \end{array}$$

where *y* and *x* and $\bar{y}$ and $\bar{x}$ are observed and modeled mean observed and modeled discharge, respectively, and *N* is the number of data pairs.

### 2.5 Land Use and Land Cover Map Preparation

The remote sensors of various spatial resolutions provided various hydrological, meteorological; land, oceanic, and vegetation types of information of the different spatial extent^[^ ^11^, ^55^ ^]^ Therefore, this research used satellite images acquired from Landsat 4–5 TM in 1989, Landsat 7 ETM+ in 2005, and Landsat 8 OLI in 2019, stored in archives for 30 years, from 1989 to 2019. Each Landsat data set has a spatial resolution of 30 m. All Landsat images of the study area have been accessed during the winter season, which has little or no cloud cover in the atmosphere, crops on the ground to minimize misclassification of particular land use, and land cover that is relatively has a similar spectral reflectance.

After successfully downloading Landsat satellite data, digital image analysis and visual image interpretation were performed. All data were projected to the Universal Transfer Mercator (UTM) projection zone 37N and the Datum of World Geodetic System (WGS 1984) to maintain data consistency during analysis. Geo‐referencing and verification of satellite imagery were conducted using a topo‐sheet obtained from the Ethiopian Mapping Agency (EMA). Thus, geo‐referencing and rectification aligning geographic data to known coordinates were performed using the topo‐sheet of 1984. Image preprocessing such as radiometric and atmospheric correction, topographic correction, image enhancement, band composite, mosaicking, and subsetting is done before land use classification. As a result, the study region has been divided into seven LULC classes based on the prevalent land use. The study area's varied LULC classes are described in **Table** **1**.

**Table 1:** Description of different LULC classes

Land use classes	General description of land use classes
Bare lands	Sediments, exposed rocks, and other areas devoid of vegetation
Built‐up areas	Settlements and roads
Farmlands	Irrigated agriculture area, fallow land, perennial crop
Grasslands	Plant communities in which grasses are dominant, shrubs are rare, and trees absent
Shrublands	Areas covered with woody vegetation mainly composed of shrubs less than 5 tall
Forest	Trees taller than 5 m and more than 0.5 hectares of land
Water bodies	Areas covered by perennial rivers, lakes, ponds, reservoirs,

The overall goal of the image classification technique is to classify all pixels in an image into land cover classes. There is no one and best image classification approach. The method depends on the nature of the analyzed data the intended application of classified data, and the available computational resources.^[^ ^55^ ^]^ As a result, because the researcher is familiar with the area of interest and has adequate knowledge of the scene, a supervised classification approach was used for this study.

The user supervises and determines the various spectral signatures associated with each class in supervised classification. This is accomplished by choosing representative samples of a known cover type, training sites, or regions. The computer algorithm then classifies the entire image using the spectral signatures from the training areas. Thus, Mahalanobis distance algorithms have been employed. The Mahalanobis distance algorithms employ statistics for each class and are direction sensitive. It is similar to maximum likelihood classification, except it assumes that all class covariances are the same, making it a faster method. Thus, Mahalanobis distance has been used because it has an advantage over other supervised classification techniques.^[^ ^56^ ^]^ For each class, thirty training samples were taken. As a result, 210 training data were created to create a thematic map of various time slices of land use classes.

## 3 Results and Discussion

### 3.1 Land Use and Land Cover Change Detection

**Figure 5:**
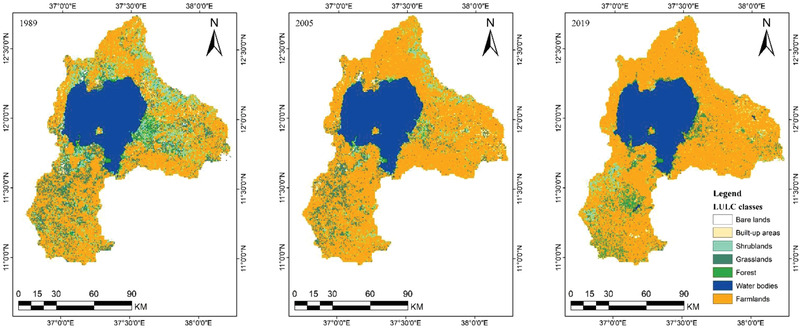
Lake Tana basin land use in the 1989, 2005, and 2019 years.

**Figure** **5** shows the basin's classified maps for 1989, 2005, and 2019. LULC maps of 2005 and 2019 have been used for accuracy assessment purposes. Therefore, the kappa coefficient and user accuracies were 85.7% and 87% for the 2005 LULC, respectively, while for the 2019 LULC, the kappa coefficient and user accuracy were 89.3% and 90.8%, respectively. The outcome indicates that the classified and ground truth classes agree. In general, the maps satisfied the requirement of minimum accuracy assessment in the following postclassification activity, such as detection of change and SWAT model input. **Tables** **2** and **3** show the confusion matrix for the Lake Tana Basin classification map in 2005 and 2019.

**Table 2:** Confusion matrix (error matrix) for Lake Tana Basin classification map in 2005

2005	Bare areas	Built‐up areas	Farmlands	Grasslands	Shrublands	Forest	Water bodies	Total	U_Accuracy	Kappa
Bare lands	13	0	1	0	0	0	0	14	0.9285	0
Built‐up areas	0	10	0	4	0	0	0	14	0.714	0
Farmlands	0	0	13	1	0	0	0	14	0.9285	0
Grasslands	0	1	1	12	0	0	0	14	0.857	0
Shrublands	0	0	0	2	12	0	0	14	0.8571	0
Forest	0	1	0	1	0	12	0	14	0.8571	0
Water bodies	0	0	0	0	0	0	14	14	1	0
Total	13	12	15	20	12	12	14	98	0	0
P_Accuracy	1	0.833	0.866	0.6	1	1	1	0	0.87	0
Kappa	0	0	0	0	0	0	0	0	0	0.857

**Table 3:** Confusion matrix (error matrix) for Lake Tana Basin classification map in 2019

2019	Bare areas	Built‐up areas	Farmlands	Grasslands	Shrublands	Forest	Water bodies	Total	U_Accuracy	Kappa
Bare lands	13	0	0	1	0	0	0	14	0.9285	0
Built‐up areas	0	11	0	2	0	1	0	14	0.7857	0
Farmlands	0	1	12	1	0	0	0	14	0.8571	0
Grasslands	0	0	0	14	0	0	0	14	1	0
Shrublands	0	0	0	1	12	1	0	14	0.8571	0
Forest	0	0	0	0	1	13	0	14	0.9285	0
Water bodies	0	0	0	0	0	0	14	14	1	0
Total	13	12	12	19	13	15	14	98	0	0
P_Accuracy	1	0.9166	1	0.736	0.923	0.8666	1	0	0.9081	0
Kappa	0	0	0	0	0	0	0	0	0	0.8928

In 30 years, the basin's farmland, built‐up areas, and forest cover increased by 2641.92, 26.66, and 338.81 km^2^, respectively. In contrast, water bodies, shrub cover areas, bare areas, and grassland decreased by 24.15, 1221.11, 143, and 1619.03 km^2^, respectively. As a result, farmland and built‐up area expansion have resulted in a decline in particular land cover (see **Table** **4**).

**Table 4:** Land use in the basin (in percent and square kilometers) in 1989, 2005, and 2019

Land use and land cover	Area in [% and km^2^]					
	1989		2005		2019	
	Km^2^	%	Km^2^	%	Km^2^	%
Bare lands	163.9	1.1	62.8	0.4	20.3	0.1
Built‐up areas	163.4	1.1	170.8	1.1	190.5	1.3
Farmlands	7527.6	50.0	9762.8	64.9	10 169.8	67.6
Grasslands	2115.2	14.1	1160.7	7.7	496.1	3.3
Shrublands	1610.0	10.7	670.5	4.5	388.8	2.6
Forest	377.9	2.5	160.7	1.1	716.7	4.8
Water bodies	3087.1	20.5	3057.2	20.3	3063.0	20.4

Table 4 shows the LULC classes in square kilometers and percentages for the basin in three periods. In contrast to the other land use/cover classes, agricultural land, and water accounted for the majority of the land use/cover classes from 1989 to 2019.

### 3.2 Calibration and Validation of SWAT+

**Figure 6:**
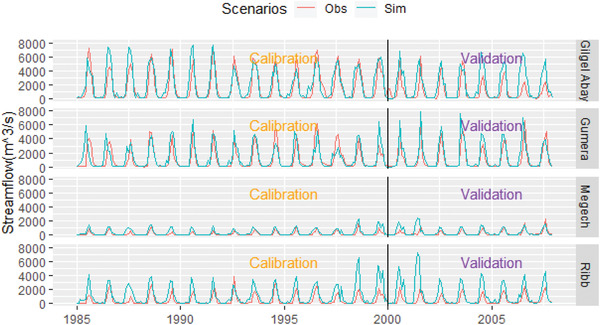
Monthly streamflow comparisons between observed and simulated data from 1985 to 2007.

Monthly streamflow data from 1985 to 1999 calibrated the SWAT+ model. Table 7 shows the parameter values used in the SWAT+ model simulations. For the warm‐up of the model, streamflow data from 1980 to 1984 were used. **Figure** **6** depicts the observed and simulated streamflow relationship during calibration (1985–1999) and validation (2000–2007). As a result, the SWAT model proved to be a valuable tool for modeling monthly streamflow in the basin instead of daily streamflow. For these reasons, monthly streamflow data have been used. The parameters chosen for model performance assessment (calibration and validation) are CN2, ESCO, AWC, ALPHA, DELAY, FLOW_MIN, REVAL_CO, and REVAL_MIN. **Table** **5** presents parameter values used for the SWAT+ model simulation. For instance, the value of groundwater delay ranges from −273.6 in Megech and 273.6 for the Ribb basin. The positive value indicates the shallow aquifer recharges to the deep aquifer, while the negative value means the deep aquifer recharges to the shallow aquifer.

**Table 5:** Parameter values used for the SWAT+ model simulation

Parameters	Default	Description	Gilgel Abay	Gumera	Megech	Ribb
CN2	±25	SCS runoff curve number for moisture condition II	−1	−1	−1	−1
GWQMN	−2000–3000	Threshold water level in shallow aquifer for baseflow	674.3389	543.1912	−2730.52	543.1912
REVAPMN	0–500	Threshold water level in shallow aquifer for revap	57.2187	57.2126	−235.42	25.8535
ALPHA_BF	0–1	Baseflow recession constant	0.7917	0.7917	−0.63.47	0.9054
GW_DELAY	−200–300	Groundwater delay time	179.2707	179.2707	−273.58	273.5805
GW_REVAP	−0.088–0	Revaporation coefficient	0.1887	0.1887	0.0100	−0.0314
SOL_AWC	±25	Available water capacity of the soil layer	1.8737	1.8737	−8.8913	−8.8913
ESCO	0–1	Soil evaporation compensation factor	0.1873	0.1873	0.3113	0.1873

The calibration and validation were conducted in the basin's four major rivers. The Gilgel Abay and Gumera rivers outlets have better calibration and validation outcomes than the Megech and Ribb rivers. The performance of the model varies from very good to satisfactory. According to ref. ^[^ ^57^ ^]^, a value of NSE between 0.0 and 1.0 is generally viewed as an acceptable level of performance, while *R* ^2^ > 0.5 is considered acceptable. On the other hand, the value of PBIAS ±25% for streamflow was rated a good simulation.^[^ ^53^, ^58^, ^59^ ^]^ Therefore, all tests performed during calibration and validation of the model fall under satisfactory to very good depending upon the type of the test. The calibration and validation results of the model confirmed that the SWAT model could be used to assess the impacts of LULC change on water balance components (**Table** **6** and Figure 6).

**Table 6:** Validation and calibration results of IPEAT

Objective function	Calibration		Rivers			
			Gilgel Abay	Gumera	Megech	Ribb
		NSE	0.712	0.60	0.51	0.62
		R^2^	0.80	0.63	0.72	0.64
		PBIAS	−15.6%	−13.5%	−24.01%	−24.5%
	Validation	NSE	0.72	0.63	0.43	0.39
		R^2^	0.80	0.77	0.50	0.55
		PBIAS	−14.53	−24.5	−25.0	−23.6

### 3.3 Impacts of LULC Changes on Hydrology at the Basin Scale

Seven major classes of multitemporal LULC: bare land, built‐up areas, farmland, grassland, shrubland, forest, and water bodies, and their spatial distribution in 1989, 2005, and 2019 are shown in (Figure 6 and **Table** **7**). During the three time periods, farmland and built‐up areas grew continuously, while shrublands, bare lands, and grasslands declined in the basin throughout the study period. For instance, farmland increased by 14.9% in 2005 compared to the 1989 proportion of farmland. Furthermore, farmland also increased by 2.7% in 2019 compared to the 2005 proportion of cropland, while grassland and shrubland decreased by 6.4% and 6.9% in 2005 compared to 1989 and 4.4% and 1.9% in 2019 compared to 2005, respectively. However, due to eucalyptus planting, the proportional coverage of forest grew from 1.1% to 4.8% from 2005 to 2019. From 1989 to 2005, built‐up areas increased by 0.05%, while the proportional extent of built‐up areas increased by 0.13% in 2019 compared to the 2005 proportion of built‐up areas. The relative size of bare lands and water bodies decreased between 1989 and 2019 (Table 7).

**Table 7:** The proportional size of LULCs, changes in LULCs, an average annual hydrological component of basin values, and changes in hydrological component values for the Lake Tana basin

Period	Bare areas [%]	Built‐up areas [%]	Farmland	Grassland [%]	Shrublands	Forest [%]	Water bodies [%]	Water yield [mm]	Surface run‐off [mm]	Baseflow [mm]	ET [mm]
1989	1.1	1.1	50	14.1	10.7	2.5	20.5	82.4	73.4	9.0	20.09
2005	0.4	1.13	64.9	7.7	4.5	1.1	20.3	83.5	74.6	8.8	19.32
2019	0.1	1.3	67.6	3.3	2.6	4.8	20.4	84.0	75.0	8.7	18.8
2005–1989	−0.7	0.05	14.9	−6.4	−6.2	−1.4	−0.2	1.1	1.2	−0.2	−0.77
2019–2005	−0.3	0.13	2.7	−4.4	−1.9	3.7	0.1	0.5	0.4	−0.1	−0.52
2019–1989	−1	0.2	17.9	−10.8	−8.7	2.3	−0.1	1.6	1.6	−0.3	−1.29

Average annual basin values for total water yield, surface runoff, baseflow, and evapotranspiration (hereafter ET) from each LULC map are summarized in Table 7. The average annual ET over the basin in 2005 was 19.32 mm, 1 and 8.8 mm in 2019, compared to the LULC baseline in 1989 in Lake Tana Basin (i.e., decreased by 0.77 and 1.29 mm, respectively, compared to 1989). Compared to the 1989 baseline period, the average annual water yield over the basin was 82.8 mm in 1989, 83.5 mm in 2005, and 84.0 mm in 2019 (i.e., increasing by 1.1, 0.5, and 1.6 mm, respectively. Similarly, the average annual baseflow steadily decreased from 9.0 mm in 1989 to 8.8 mm in 2005 (a decrease of 0.2 mm). The comparison of surface runoff variations and changes in LULC showed that the rise in average annual basin‐scale surface runoff could mainly be due to the expansion of farmland and the shrinking of grasslands and shrublands from 1989 to 2019 (Table 7).

The expansion of agricultural land in the Lake Tana basin has replaced grassland and shrublands. In contrast to grassland and shrub cover areas, the addition of farmland reduces soil infiltration rate, and baseflow increases surface runoff. Growth in agriculture can also change the local water balance at the expense of grasslands and shrublands. A comparison of water yield changes and LULCs in the Lake Tana basin (Table 7) reveals that the increase in water yield from 1989 to 2019 is due to a consistent increase in agricultural land and a concurrent decrease in shrublands.

### 3.4 Lake Tana Basin Lateral Flow, Surface Runoff, Evapotranspiration, and Water Yield

**Figure 7:**
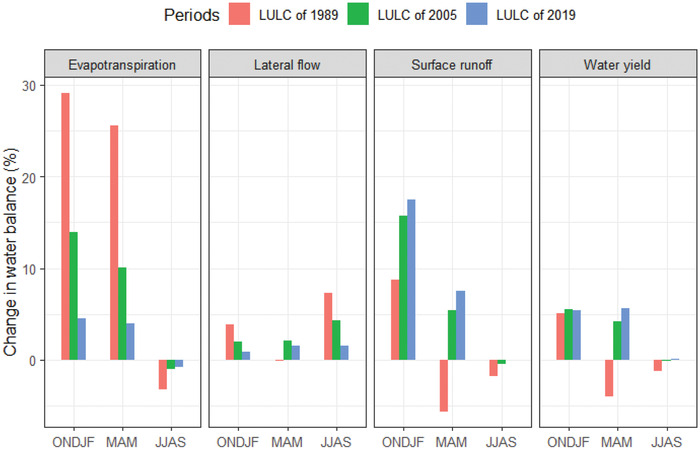
Simulated impact of changing land use on seasonal water balance components in the Lake Tana basin in 1989, 2005, and 2019.

**Figure 8:**
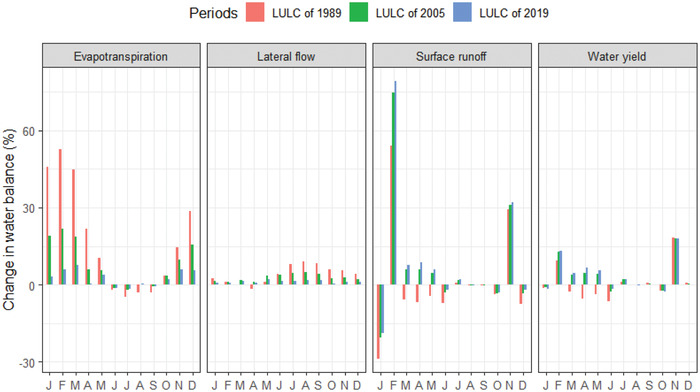
Simulated impact of changing land use on monthly water balance components in the Lake Tana basin in 1989, 2005, and 2019.

**Figures** **7** and **8** show the mean monthly and seasonal change of lateral flow under the LULC of 1989, 2005, and 2019. Under the LULC of 1989, the highest lateral flow change was observed, followed by the LULC change of 2005, while under the LULC change of 2019, the lowest lateral flow change was observed. Unlike other seasons and months, lateral flow is likely to decline under the 1989 LULC in April and May. The most remarkable change in lateral flow during June, July, August, and September was observed relative to other months.

Figures 7 and 8 depict the mean monthly and seasonal change in evapotranspiration in 1989, 2005, and 2019 under the LULC. During the summer season under the LULC of 1989, 2005, and 2019, monthly evapotranspiration is likely to decrease during June, July, August, and September (JJAS). The highest evapotranspiration was observed under the three LULC changes in 1989, followed by LULC in 2005. On the other hand, during 2019 LULC, the lowest evapotranspiration has been recorded. During the winter months, when most crops and plants die are known to have only minimal canopy coverage. Still, the range increases in spring through summer from October to April, and the mean monthly evapotranspiration of the 1989 LULC transition was more significant than the LULC change between 2005 and 2019.

On the other hand, from May to September, the average monthly evapotranspiration in 1989 was lower than 2005 and 2019. Figures 7 and 8 show the seasonal and monthly surface runoff change under the land use of 1989, 2005, and 2019. In January, June, October, and December, the monthly surface runoff decreased under land using 1989, 2005, and 2019. Furthermore, surface runoff of the basin is also declining during the summer season of the basin. Figures 7 and 8 also indicate the simulated impacts of land‐use change on the water yield of the basin. Thus, water yield changes under land use in 1989, 2005, and 2019 have a relatively similar pattern to surface runoff of the basin. Hence, water yield declined during the January and October months of the basin under land‐use changes in 1989, 2005, and 2019.

### 3.5 Spatial Distribution of Hydrological Components

#### 3.5.1 Spatial Distribution of Evapotranspiration

**Figure 9:**
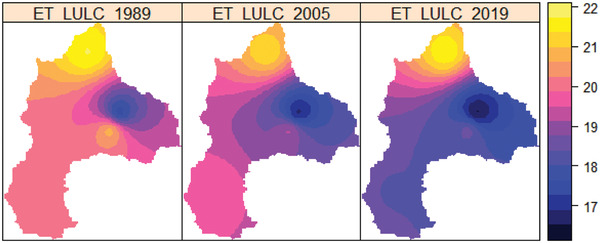
The spatial distribution of the basin's mean annual evapotranspiration (mm) in 1989, 2005, and 2019.

The mean annual evapotranspiration was indicated in **Figure** **9** for the 1989, 2005, and 2019 years. As shown in Figure 9, the spatial variation of evapotranspiration varies from 17 to 22 mm in 1989, 2005, and 2019 LULC, respectively. The highest evapotranspiration was found in the basin around and within the Megech River (North). In contrast, the lowest evapotranspiration was found in the Ribb River (East) under LULC changes in 1989, 2005, and 2019. The basin of the Megech River is relatively mountainous and has a high wind speed, which is why greater evapotranspiration has been observed. On the other hand, mild evapotranspiration has been found along the Gilgel Abay (Southern) and Gumera (Eastern) Rivers watersheds.

#### 3.5.2 Spatial Distribution of Lateral Flow

**Figure 10:**
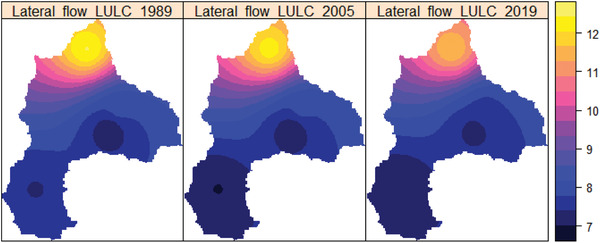
The spatial distribution of the basin's mean annual lateral flow (mm) in 1989, 2005, and 2019.

In **Figure** **10**, the spatial variation of the lateral flow was portrayed 1989, 2005, and 2019. The spatial variation of the baseflow varies from 7 to 12 mm in 1989, 2005, and 2019 LULC, respectively, as shown in Figure 10. The highest lateral flow was observed within the Megech River watershed in the basin. In contrast, the lowest baseflow was found in the parts of Gilgel Abay, Ribb, and Gumera Rivers watershed under the land‐use change in 1989, 2005, and 2019. On the other hand, mild baseflow has been observed around the northern part of Lake Tana.

#### 3.5.3 Spatial Distribution of Surface Runoff

**Figure 11:**
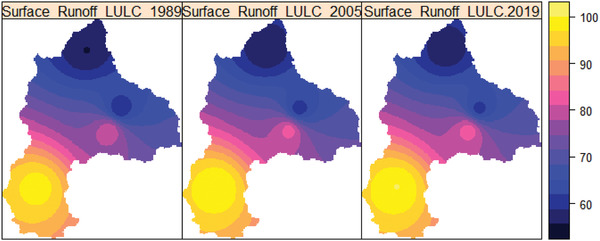
The spatial distribution of the basin's mean annual surface runoff (mm) in 1989, 2005, and 2019.

**Figure** **11** depicts the spatial variation of surface runoff in 1989, 2005, and 2019. As shown in Figure 11, in 1989, 2005, and 2019 LULC, the spatial variation of surface runoff varies from 55 to 100  mm, respectively. The Gilgel Abay River's watershed was found to have the highest surface runoff in the basin. In contrast, the lowest surface runoff was found in Megech, whereas a mild surface runoff in the Ribb and Gumera River watersheds.

#### 3.5.4 Spatial Distribution of Water Yield

**Figure 12:**
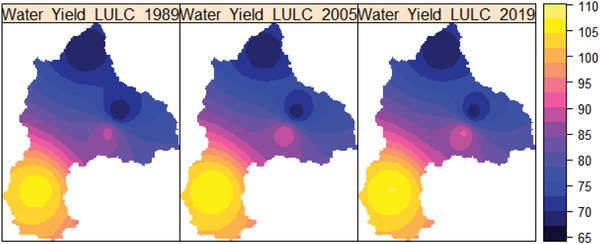
The spatial distribution of the basin's mean annual water yield (mm) in 1989, 2005, and 2019.

**Figure** **12** depicts the spatial variation of water yield in 1989, 2005, and 2019. As shown in Figure 12, the spatial variation of water yield varies from 67 to 107.5 mm in 1989, 2005, and 2019 LULC. Under LULC change in 1989, 2005, and 2019, the highest water yield in the basin was found around and within the watershed of the Gilgel Abay River, the lowest water yield in Megech, and mild water yield in the Ribb and Gumera watersheds.

## 4 Discussion

Understanding the effects of changing land use on the process of hydrology is a prerequisite for developing and implementing long‐term land and water resource management plans. The SWAT model was used in this study to analyze the effects of changing land use on the hydrology of the Lake Tana basin. Forest land cover declined mainly due to farmland and built‐up area expansion. This is followed by unsustainable methods for integrated watershed management and population growth within the basin. The essential factors of changing land use are lack of market and financial facilities, poverty, land tenure, and population growth. According to this finding, evapotranspiration has tended to grow from 1989 to 2019 land use due to increased forest cover. In line with these findings,^[^ ^60^ ^]^ indicated that changes in land use have a more significant impact on evapotranspiration than climatic change. An increase in streamflow, lateral flow, and evapotranspiration in the basin will significantly impact the water supply. The basin's water supply is affected by water supply abstractions.^[^ ^61^ ^]^ In general, the study area's land use caused an increase in mean annual surface runoff and water yield from 1989 to 2019. However, the basin's mean annual baseflow and evapotranspiration declined in 2019 compared to the area's 1989 and 2005 land use. The result of this finding is relatively consistent with different studies conducted in the study area subwatershed level and a river basin. In line with this^[^ ^62^ ^]^ indicated that land‐use changes that affected hydrological components in the basin were the expansion of cultivation land, built‐up area and grassland, and decline in natural forests. Similarly,^[^ ^63^ ^]^ also indicated that cultivation and afforestation have a consistent impact on the entire range of discharges, especially low flows.

For instance,^[^ ^64^ ^]^ reported that increasing the size of farmlands and bare lands caused higher seasonal and annual sediment yield and streamflow volumes. A study in the Andassa watershed by^[^ ^14^ ^]^ also revealed that LULC change from 1985 to 2015 had increased annual flow, water yield, surface runoff, and wet season flow but decreased groundwater flow, dry season flow, lateral flow, and evapotranspiration. Another study by^[^ ^65^ ^]^ also indicated that the mean wet monthly flow increased, while dry average monthly flow decreased from 1985 to 2011 land cover due to an expansion of farmlands and built‐up area and a decrease of grassland and forest. Similarly,^[^ ^66^ ^]^ indicated that the mean wet and dry monthly flow for 2010 land cover compared to 1986 land cover increased and decreased, respectively, due to an increase in farmlands and residential areas and a decrease in forest and grassland in Ketar Watershed of Lake Ziway catchment area. Between 2000 and 2010, the conversion of grassland to barren or sparsely vegetated land reduced available energy for evapotranspiration, resulting in increased runoff in China's Heihe River Basin (Deng et al. 2015). Another study^[^ ^67^ ^]^ indicated that land cover alterations resulted in a considerable decrease in runoff depth values in the Al‐Baha region of Saudi Arabia. In general, increased farmland and built‐up patterns are the primary cause of increased surface runoff and water yield in the basin. A drop in lateral flow would hurt the basin's agricultural system. Hence, because of the decrease in lateral flow, the basin's soil water would decrease, affecting water supplies for crops.

## 5 Conclusion

This study detects the land‐use change in the Lake Tana basin for 30 years in three‐time slices (i.e., 1989, 2005, and 2019). After detecting the land‐use changes, their impacts on the hydrological processes of the Lake Tana Basin were investigated using the SWAT model. Farmlands, residential, and forest areas in the basin have increased by 2641.92, 26.66, and 338.81 km^2^ over the last 30 years, respectively, while water bodies, shrublands, grasslands, and bare lands have decreased in the basin by 24.15, 1221.11, 1619.03, and 143 km^2^, respectively due to expansion of farmlands and residential areas in the basin. During the three‐time slices, farmlands, and the built‐up lands continuously increased, while shrublands, grasslands, and bare areas declined in the basin during the study period. The finding showed that LULCs in 2005 and 2019 improved surface runoff, water yield, and streamflow relative to baseline LULC maps in 1989 in the entire basin‐scale and the sub‐basin scale of Lake Tana due to an increase in farmland and built‐up area. In line with this find,^[^ ^68^, ^69^ ^]^ reported that the watershed's hydrological response is closely related to land cover patterns. In the Lake Tana Basin, baseflow and evapotranspiration of hydrological components decreased between 2005 and 2019 compared to 1989 baseline maps. Using spatial tools, the spatial distribution of hydrological components was also analyzed. The result suggested that the highest surface runoff and water yield were located within and around the watershed of Gilgel Abay. At the same time, the lowest was found within and around the watershed of Megech.

On the other hand, inside and around the watershed of Gumera and Ribb, mild water yield and surface runoff have been identified. The spatial distribution of evapotranspiration and baseflow showed the highest value in the Megech watershed, while the lowest was seen around the watershed of Gilgel Abay. In general changing land use in the basin is a critical issue that disturbs the natural flow of water. To this effect, appropriate land use management activities should be implemented to protect and maintain natural resources.

## Conflict of Interest

The authors declare no conflict of interest.

## Data Availability

The data that support the findings of this study are available from the corresponding author upon reasonable request.
